# Understanding *P. falciparum* Asymptomatic Infections: A Proposition for a Transcriptomic Approach

**DOI:** 10.3389/fimmu.2019.02398

**Published:** 2019-10-15

**Authors:** Kelvin M. Kimenyi, Kevin Wamae, Lynette Isabella Ochola-Oyier

**Affiliations:** ^1^KEMRI-Wellcome Trust Research Programme, CGMRC, Kilifi, Kenya; ^2^Centre for Biotechnology and Bioinformatics, University of Nairobi, Nairobi, Kenya; ^3^Pwani University Bioscience Research Centre, Pwani University, Kilifi, Kenya

**Keywords:** asymptomatic infection, malaria, immunity, cytokines, RNA-seq

## Abstract

Malaria is still a significant public health burden in the tropics. Infection with malaria causing parasites results in a wide range of clinical disease presentations, from severe to uncomplicated or mild, and in the poorly understood asymptomatic infections. The complexity of asymptomatic infections is due to the intricate interplay between factors derived from the human host, parasite, and environment. Asymptomatic infections often go undetected and provide a silent natural reservoir that sustains malaria transmission. This creates a major obstacle for malaria control and elimination efforts. Numerous studies have tried to characterize asymptomatic infections, unanimously revealing that host immunity is the underlying factor in the maintenance of these infections and in the risk of developing febrile malaria infections. An in-depth understanding of how host immunity and parasite factors interact to cause malaria disease tolerance is thus required. This review primarily focuses on understanding anti-inflammatory and pro-inflammatory responses to asymptomatic infections in malaria endemic areas, to present the view that it is potentially the shift in host immunity toward an anti-inflammatory profile that maintains asymptomatic infections after multiple exposures to malaria. Conversely, symptomatic infections are skewed toward a pro-inflammatory immune profile. Moreover, we propose that these infections can be better interrogated using next generation sequencing technologies, in particular RNA sequencing (RNA-seq), to investigate the immune system using the transcriptome sampled during a clearly defined asymptomatic infection.

## Introduction

Malaria is a significant public health burden with an estimated 219 million new cases and 435,000 deaths reported in 2017 (1). Nearly half of the world's population is at risk of contracting malaria, with tropical and subtropical areas showing the highest prevalence (2). Malaria infection is caused by protozoan parasites of the genus *Plasmodium* that affect humans. *P. falciparum* is globally the most deadly and is the most prevalent parasite in Africa (3). *P. falciparum* malaria ranges from severe to uncomplicated or mild and to the poorly understood asymptomatic infections. Such diverse outcomes are due to the intricate interplay between factors derived from the human host, parasite, and environment (4). At the genomic level, differences in gene expression by the host during host-parasite interactions may account for the various clinical manifestations (5). Specifically, gene pathways that regulate cytokine signaling and complement regulation as well as the production of immunoglobulins have been implicated (6). A strong pro-inflammatory response has been associated with an increased risk of febrile malaria, severe malaria anemia (7) or cerebral malaria (8), while a weak response has been associated with asymptomatic infection (9). Hence, the balance between pro-inflammatory and anti-inflammatory cytokine production appears to be important in influencing the outcome of malaria infections. Identification of markers that can diagnose the clinical manifestations of *P. falciparum* infections, in addition to symptoms, is important in predicting prognosis and directing treatment strategies.

Malaria infections are mainly characterized by a recurrent cycle of fever and chills. Other symptoms include vomiting, shivering, convulsions, and anemia caused by hemolysis (10). In some cases, these symptoms are not observed, and the infection is described as asymptomatic in individuals without a recent history of antimalarial treatment (11). Once an individual is infected with the parasite, immune factors are tasked with reducing parasite numbers, i.e., anti-parasite immunity, and preventing manifestation of clinical symptoms, anti-disease immunity. In asymptomatic individuals, immunity is skewed toward anti-disease rather than anti-parasite immunity. The mechanisms behind this phenomena are still unclear and more studies are required to understand how anti-disease immunity is induced and its potential for application in vaccine development (12).

## Defining Asymptomatic *Plasmodium falciparum* Infections

The study of asymptomatic infections is still hampered by the lack of standard criteria for defining these infections (4, 11). This is due to the wide range of definitions that complicates the comparison of results across studies (Table 1). The most basic definition seems to be the presence of parasitemia and the absence of malaria symptoms, mainly fever (axillary temperature <37.5°C) (14, 19, 20). This definition is ambiguous and most studies have modified it by incorporating strict inclusion criteria. Laishram et al. (4) summarized the diagnostic criteria used to define asymptomatic individuals in different studies and made several recommendations. They suggested the use of longitudinal follow ups, quantifying parasitemia rather than reporting its presence or absence and the use of PCR to identify asymptomatic infections in a population (4). Since then, the criteria have improved by incorporating the latest advancement in PCR, the loop-mediated isothermal amplification (LAMP), biomarkers to detect the parasite and use of cohorts that ensure reliable information about clinical history and follow ups. This allows for the exclusion of those who experienced symptoms in the recent past and then sought treatment. However, there is no consensus on the duration of history and it ranges from 2 weeks to 1 month (5, 14, 20). The longitudinal follow-ups after diagnosis reduces the chances of “false” asymptomatic parasitemia that are defined during *P. falciparum* incubation toward a clinical outcome. The duration of follow-up varies depending on whether the study is interested in asymptomatic parasitemia or the eventual symptomatic outcome (5, 14). The method for asymptomatic parasitemia diagnosis is also important. Microscopy, with a detection threshold of ~50 parasites μl^−1^, may miss subpatent infections, while others use PCR whose sensitivity can extend to below one parasite μl^−1^ (21, 22). Studies in Kenya, Uganda and Brazil have reported a significantly high prevalence of asymptomatic parasitemia, as much as 6–7 times higher, using PCR when compared to microscopy (13, 23, 24). PCR has also helped to identify individuals with low-density parasitemia in low-transmission settings that were previously missed by microscopy (25). Although the use of PCR is technical and expensive, making it unrealistic in most field studies, it is important in improving the accuracy of diagnosing asymptomatic parasitemia (26). Interestingly, LAMP has been shown to accurately detect sub-microscopic asymptomatic *Plasmodium* infections (27). LAMP is cheap and easy to implement in a field setting as it does not require a thermocycler machine like PCR. In addition, several biomarkers such as lactate dehydrogenase, hemozoin and, in particular, Histidine-Rich Protein 2 that is utilized in rapid diagnostic tests (RDTs), have been used to diagnose malaria (28, 29). Hemozoin is an important metabolite of hemoglobin digestion by the malaria parasite and is associated with pathogenesis as well as inducing immunity to malaria (30–32). A hemozoin sensing assay has recently been shown to be 20 times more sensitive than RDTs in diagnosing *Plasmodium* species (33). It could be applied as a point of care test and more importantly in screening populations for asymptomatic individuals with submicroscopic parasitemia (33). More efficient diagnostic techniques are needed to effectively detect asymptomatic infections in various settings to improve the quality and reliability of data used in studying asymptomatic infections. Table 1 outlines the different criteria used in transcriptomics studies to define asymptomatic *P. falciparum* infections.

**Table 1:** Examples of inclusion criteria used to define asymptomatic individuals in transcriptomic studies.

**Country, year**	**Criteria for identifying asymptomatic cases**	**Study subjects (sample size)**	**Follow-up protocol, duration**	**References**
Cameroon, 2009	Positive thick blood smear and afebrile. No history of fever and antimalarial treatment in the previous 1 and 2 weeks, respectively, at the time of mass screening	Children <12 years (13)	No follow-up	(5)
Mali, 2011	PCR-detected *P. falciparum* and no fever. No history of antimalarial or immunosuppressive medication in the last 30 days and helminths	Individuals >13 (5)	Bi-weekly and weekly surveillance for *Plasmodium infection* and malaria episode, respectively	(14)
Gabon, 2005	Thin and thick blood smear and no clinical symptoms	Children 0.5–6 years (ND)	Follow up for 5 consecutive days	(6)
Uganda, 2007–2008	Blood smear and no fever	Children 4–5 years (15)	Follow up for 7 days	(16)
Mali, 2006	Not defined	5–13 years (17)	Healthy baseline before the malaria season, 7 or 14 days after treatment of their first malaria episode of the ensuing malaria season, and a subset of children followed up to the 6-month dry season	(18)

## Understanding the Risk of Developing Febrile Infections

**Figure 1:**
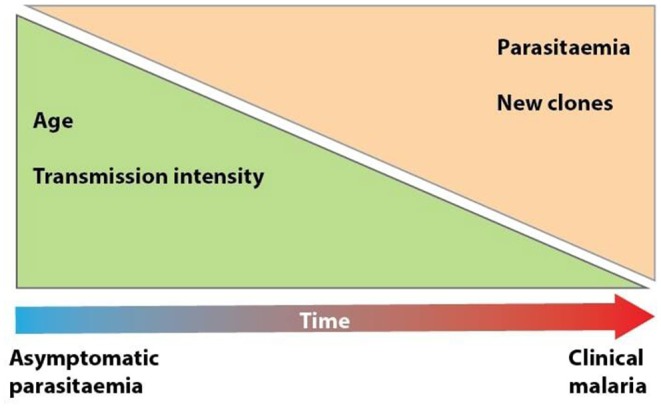
Progression from asymptomatic to clinical malaria. Developing clinical malaria following an asymptomatic infection is influenced by host, parasite, and environmental factors. As individuals age and transmission intensity increases, the risk of developing clinical malaria decreases. This is primarily due to the development of acquired immunity in malaria endemic areas where exposure to repeated infection is common. An increase in parasitemia increases the risk of developing clinical malaria, while acquisition of new parasite clones increases the risk of developing symptoms due to lack of protective immunity against the new clones.

Asymptomatic infections can act as precursors to malaria illness (34). Mass drug administration (MDA) has been suggested as an effective way of treating chronic asymptomatic infections (35, 36). However, this may interfere with the immunity maintained by these infections, thus increasing the risk of developing clinical malaria in asymptomatic individuals (37). A study in Mali treated chronic asymptomatic individuals at the end of the dry season, followed them up during the subsequent rainy season and reported that treatment of asymptomatic infections is unlikely to influence the subsequent risk of developing clinical malaria (17). Similar findings were also reported in Burkina Faso (38). A risk-benefit analysis is required to determine the tradeoffs to inform the public health impact of MDA on asymptomatic infections. The possibility of developing febrile malaria among asymptomatic carriers has been shown to vary due to transmission intensity and age (Figure 1) (20, 39). A study in Kenya compared the risk of developing febrile malaria among children (0–15 years) who were uninfected with *P. falciparum* and those with asymptomatic parasitemia. In lower transmission areas, asymptomatic parasitemia was linked to an increased risk of febrile malaria in children of all ages, while in moderate to high transmission areas, asymptomatic parasitemia was linked to a reduced risk of febrile malaria in children above 3 years (20). High asymptomatic parasitemia densities (≥2,500 parasites μL^−1^) and every 10-fold increase in parasite density have been associated with an increased risk of developing febrile malaria, probably due to the underlying reduced host immunity (20, 39).

In addition, the transition from asymptomatic to symptomatic malaria may be influenced by the presence of new parasite genotypes to which an individual has not been previously exposed in the preceding asymptomatic infection, and hence they are likely to lack protective immune responses to the new genotype (40–42). *P. falciparum* infections in malaria endemic areas, primarily in sub-Saharan Africa, are characterized by the co-circulation of multiple *P. falciparum* clones in acute and persistent infections (43–47). This phenomenon is termed complexity of infection (COI). The presence of multiclonal infections has been associated with an increased range of anti-merozoite antibody responses. Together, COI and anti-merozoite antibody responses have been associated with a reduced risk of clinical malaria (48). Carriage of certain merozoite surface protein (*msp*) 1 genotypes have been associated with different clinical manifestations of malaria. In Nigerian children, *msp1* K1 and MAD20 alleles were associated with asymptomatic malaria and a minimal risk of becoming febrile (49). Similarly, using the *msp2* locus, the FC27 genotype was more prevalent in asymptomatic than in symptomatic Nigerian children (50). Other studies have shown that an increase in COI in children with asymptomatic infections is associated with an increased risk of febrile malaria in younger children, a lower risk in older children (51, 52) and in some cases, age had no influence (41, 53). Such conflicting findings on the role of COI in predicting febrile malaria could be due to methodology.

The traditional method for characterizing *P. falciparum* diversity uses nested-PCR with gel electrophoresis to detect polymorphisms in *msp1, msp2* and glutamine rich protein (GLuRP) (54) and recently by capillary electrophoresis (55). This technique has been shown to be limited in the number of variants detected within an individual infection (56, 57), is insensitive to less abundant variants and is not quantitative for relative proportions of circulating variants (57). Amplicon deep-sequencing overcomes these challenges as it is more sensitive than capillary electrophoresis in detecting minority clones and is able to quantify individual *P. falciparum* clones (58). This technique also uses less DNA since adequate amounts of amplicons are obtainable directly from dried blood spots used for screening large sample sizes (59). With increased sensitivity and number of samples that can be genotyped, deep-sequencing promises to increase our understanding of the dynamics of *P. falciparum* COI and how this relates to the outcomes of febrile malaria. An understanding of how host immunity mediates the development of febrile malaria in asymptomatic carriers is required to better understand these infections.

## Immunity to Malaria

Individuals residing in malaria endemic zones often harbor asymptomatic infections and are clinically immune due to exposure to multiple genetically complex *P. falciparum* infections over time (60). Children who have experienced repeated malaria episodes have a modified immune system (26, 61, 62) that is for instance characterized by an increased production of immunoregulatory cytokine IL-10 and activation of neutrophils, B cells and CD8^+^ T cells (63). The immune system is involved in controlling disease outcome as exhibited by the fact that the parasitemia tolerated in high transmission settings is higher than that causing fever in low transmission settings (64). The chances of an infection being asymptomatic increases with age as repeated exposure to malaria leads to the development of partial anti-disease immunity (65). Unfortunately, the immunity developed is not sterile, only suppressing but not eliminating the infection leading to disease tolerance and asymptomatic infection (66, 67). This immunity may also be lost due to a lack of continuous exposure to the parasite, resulting in elevated pro-inflammatory responses and subsequently a high risk of illness (68).

## Immunomodulation in Asymptomatic Malaria

Modulation of immune responses has been associated with different clinical malaria manifestations (66, 69). The immune responses are mediated by cytokines that regulate inflammation and are thus involved in protective immunity. These cytokines include interferon gamma (IFN-γ), tumor necrosis factor alpha (TNF-α) and IL-12 (70, 71). Overstimulation of the immune system leading to excessive production of these cytokines and activation of immune cells is detrimental to the host as they are likely to cause severe malaria symptoms through unknown mechanisms (72, 73). However, anti-inflammatory cytokines including IL-10, IL-27 and tissue growth factor beta (TGF-β) were shown to be involved in dampening pro-inflammatory cytokines, thereby minimizing disease severity (71, 74). The presence of anti-inflammatory cytokines, especially IL-10, suppresses parasite clearance, hindering the development of anti-parasite immunity, and clinical malaria (69), while promoting the development of asymptomatic infections (18). Elevated levels of IL-10 has also been linked to asymptomatic infections in pregnant women (75).

The production of anti-inflammatory cytokines has been shown to increase with repeated exposure to malaria, resulting in asymptomatic infection (18). Conversely, a lack of continuous exposure in historically exposed individuals can lead to the loss of anti-disease immunity (68, 76). This was exhibited by an increased production of pro-inflammatory cytokines and the proliferation of CD4^+^ T cells (68). A study in Ugandan children revealed that the production of cytokines by CD4^+^ T cells is influenced by prior exposure to malaria infections. CD4^+^ T-cells in more exposed children were shown to produce higher levels of IL-10, while those in less exposed children produced higher levels of TNFα, hence promoting inflammation. The lack of TNFα production was associated with asymptomatic infections (9). A transcriptomic study in Mali described the activation of pro-inflammatory cytokine (IFN-γ, TNF, and IL-1β) production as being influenced by prior exposure to malaria, with asymptomatic infections having the least activation of these cytokines (14). Another study in Uganda revealed that frequent exposure to malaria infection causes decreased levels of pro-inflammatory cytokine (IFN-γ, TNF) producing Vδ2^+^ γδ T cells and increased expression of immunoregulatory genes potentially dampening symptom development upon subsequent infections (16). In addition to exposure, age differences have also been suggested to modulate the immune system, with older children having lower anti-inflammatory and pro-inflammatory responses as compared to younger children (15). Lower levels of regulatory T cells (T regs) has also been observed in asymptomatic compared to symptomatic individuals (77, 78). Additionally, high levels of T regs have been associated with increased parasitemia, TGF-β production and the development of clinical symptoms (77, 79, 80). Lower T reg levels, on the other hand, may result in a decreased risk of developing symptoms, which translates to anti-disease immunity (78).

Unlike in asymptomatic infections, the Fulani ethnic group, who have reduced susceptibility to *P. falciparum* infection compared to sympatric tribes, have a higher ratio of pro-inflammatory to anti-inflammatory cytokines (81). The higher levels of pro-inflammatory cytokines have been implicated in causing the reduced symptomatic cases and parasite densities. In a transcriptomic study of their monocytes, increased upregulation of gene pathways involved in the production of pro-inflammatory cytokines in uninfected Fulani was observed, potentially priming the immune system to respond more effectively to *P. falciparum* infections (82). Thus, it appears that a balance between inflammatory and regulatory cytokines is important in achieving anti-disease immunity.

**Figure 2:**
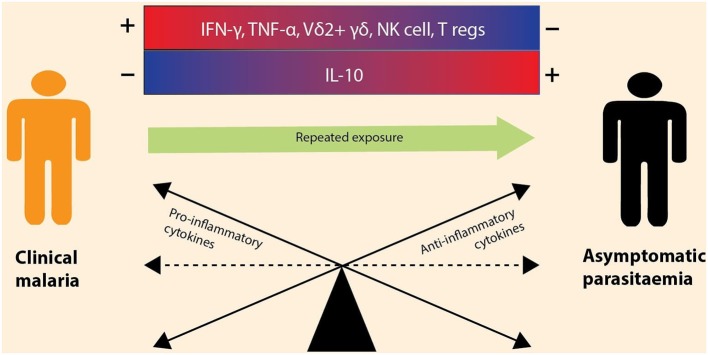
Immune modulation during asymptomatic vs. symptomatic malaria infections. The outcome of a malaria infection is influenced by the balance between anti-inflammatory and pro-inflammatory cytokines. Clinical malaria is the result of elevated (+) production of pro-inflammatory cytokines (e.g., IFN-γ, TNF-α) and increased levels of immune cells (e.g., Vδ2^+^ γδ, NK cells, and T regs) and the downregulation (–) of anti-inflammatory cytokines (e.g., IL-10). However, with repeated malaria exposure, the immune balance shifts toward an increased production of anti-inflammatory cytokines, leading to asymptomatic infection. The cytokines are encoded by immune genes, thus differential expression of these genes depicts that there is a balance between anti-inflammatory and pro-inflammatory cytokines.

Antibodies also play a significant role in malaria protection. Seminal studies in monkeys and humans reported a reduction in fever and parasitemia following the passive transfer of serum or IgG antibodies from immune to non-immune subjects with acute malaria (83, 84). However, antibody responses to malaria seem to be short lived as exposure to malaria may not lead to the production of sufficient antigen-specific memory B cells (85). In contrast, a study on Swedish travelers previously treated for malaria maintained long-lasting memory B cells for 16 years without subsequent exposure (86). Higher titers of antigen-specific IgG have been observed in asymptomatic individuals compared to individuals with other malaria outcomes (87–89). Furthermore, high antigen-specific antibody responses were associated with high levels of IL-10 and IFN-γ in Gabonese children with asymptomatic *P. falciparum* infection, suggesting that the antibody response may exert protective immune mechanisms (90). Further studies of immune cells and cytokines (Table 2) are necessary to understand the mechanisms underlying immunomodulation and how this can be applied to confer malaria protection. It is evident that there is a complex interplay of various components of the immune system (Figure 2), and one way of potentially interrogating this complexity is through an “omics” approach.

**Table 2:** A list of selected cytokines and immune cells showing their levels as reported in studies from Africa comparing malaria clinical outcome.

	**Levels**	**Study site**	**Clinical comparison**	**References**
IL-10	High	Uganda	Asymptomatic/symptomatic	(9)
	High	Ghana	Asymptomatic/uninfected	(75)
	High	Mali	Asymptomatic/febrile	(18)
	High	Gabon	Asymptomatic/mild	(90)
IFN-γ	Low	Uganda	Asymptomatic/febrile	(9)
	High	Gabon	Asymptomatic/mild	(90)
TNFα	Low	Uganda	Asymptomatic/febrile	(16)
	Low	Uganda	Asymptomatic/symptomatic	(9)
Tregs	Low	Uganda	Asymptomatic/febrile	(77)
	Low	Ghana	Asymptomatic/symptomatic	(78)
Vδ2^+^ γδ T cells	Low	Uganda	Asymptomatic/febrile	(16)
Natural killer cells	Low	Kenya	Asymptomatic/uninfected	(26)

To add to this already complex immune process, co-endemicity of *P. falciparum* and helminths is very common in the tropics resulting in increased chances of co-infection (91). Interactions between the two parasites alter immune responses, thus influencing susceptibility to clinical malaria (92, 93). There are conflicting reports on the outcome of these interactions as some studies have reported enhanced severity (94, 95), others reduced severity (96, 97) yet others have revealed no association between helminth co-infection and malaria outcome (98, 99). These observations may be due to differences in the co-infecting helminth species (100), the host's level of immunity to *P. falciparum* (101) and differences in study design (91). Various helminths elicit different immune responses that have an impact on the immunopathology of malaria as a result of the imbalance between pro-inflammatory and anti-inflammatory cytokines (102). However, there are contradictory reports on cytokine profiles resulting from helminths and *P. falciparum* co-infection. Co-infection with *Schistosoma haematobium* in Senegal was shown to offset this balance in an age-dependent manner in children and adults when compared to *P. falciparum* mono-infections. Co-infected children had higher levels of pro-inflammatory cytokines, IFN-γ and TNF-α, and were more likely develop febrile malaria, while co-infected adults had higher levels of similar pro-inflammatory cytokines as well as anti-inflammatory cytokines IL-10 and TGF-β and were more protected from malaria morbidity (103). In Uganda, IL-10 and IL-6 levels were elevated during *P. falciparum* and soil-borne helminth co-infection, while the level of TGF-β was reduced (104). The level of IL-10 was also elevated in asymptomatic *P. falciparum* Malian patients with filaria co-infection while IFN-γ, TNF-α, and IL-17 levels were reduced (105). There is growing evidence that *P. falciparum* and helminth co-infections have a profound effect on the host immune system, preventing the immune clearance of either parasite over the other. Although modulation of pro-inflammatory and anti-inflammatory cytokines responses has been suggested as a possible mechanism, more studies are needed to understand the roles of these cytokines and how other aspects of the immune system are involved.

## Asymptomatic *P. falciparum* Transcriptomics Studies

A number of studies have examined the transcriptomics of asymptomatic *P. falciparum* infections and revealed interesting findings that add to the current phenotypic knowledge of these infections. The first study compared parasite gene expression patterns between 18 cerebral and 18 asymptomatic malaria infections in Cameroonian children using microarrays. The major difference was observed in genes coding for exported proteins, transcriptional factor proteins, proteins involved in protein transport, variant surface antigen (VSA) proteins such as *P. falciparum* erythrocyte membrane proteins (PfEMP) and repetitive interspersed family (RIFIN) (5). A more recent study in Gabon compared the host transcriptomic profiles between children with uncomplicated, asymptomatic, severe and cerebral malaria and identified 36 genes, among 4,643 transcripts, which are specifically regulated during asymptomatic infections. These genes are involved in nucleotide binding and RNA processing alluding to gene regulation via chromatin remodeling as a potential mode of maintaining asymptomatic infections (6). Chromatin remodeling changes the chromatin architecture, making condensed genomic DNA accessible to the transcription proteins and thereby regulating gene expression. This was shown to influence activation of immune cells such as monocytes and macrophages (106). It is hypothesized that during asymptomatic infections, chromatin remodeling decreases the expression of immunoglobulin genes leading to reduced antibody mediated responses (6). An opposite effect of chromatin remodeling is observed in the Fulani ethnic group. Chromatin remodeling may have resulted in stronger transcriptional activity in the monocytes leading to a pro-inflammatory state and reduced susceptibility to malaria infections when compared to sympatric ethnic groups (82). Pre- and post-infection profiles of Malian adults who were either febrile, asymptomatic or naïve were compared. Interestingly, asymptomatic individuals had the least transcriptional changes in gene pathways that are regulated by pro-inflammatory cytokines, IFN-γ, TNF, and IL-1β (14). This was probably caused by the downregulation of genes encoding pro-inflammatory cytokines, as observed in their lower levels in individuals with frequent exposure to *P. falciparum* in Mali (18). Chronic exposure to *P. falciparum* was shown to cause increased expression of immunoregulatory genes in Vδ2^+^ T cells in Ugandan children (9). The results from the various transcriptomic studies in asymptomatic infections tend to agree that gene regulation mechanisms, either transcription factors or changes in the chromatin structure, may be involved in regulating inflammatory mechanisms that maintain malaria infections in the asymptomatic state. This can be confirmed by studying chromatin accessibility of gene regions that encode inflammatory regulators. Various techniques have been developed to assess genome-wide chromatin accessibility, with the Assay for Transposase-Accessible Chromatin using sequencing (ATAC-seq) proving to be the most effective (107). This technique has been applied in examining chromatin accessibility in *P. falciparum* and revealed insights into how the accessibility of specific transcriptome regulatory regions, i.e., promoter regions, are directly associated with an abundance of their corresponding transcripts (108, 109). The identification of regulatory regions that regulate expression of inflammatory genes in asymptomatic infections is important, as it would improve our understanding of how anti-disease immunity is maintained and reveal regions that can be targeted to achieve anti-parasite immunity and potentially, sterilizing immunity.

## Future Perspectives For Asymptomatic Studies

Asymptomatic malaria appears to be driven by host immunity, such that following multiple exposures to symptomatic infections an individual's immune system is potentially primed to control symptoms. Initially, at a young age, individuals in malaria endemic areas are likely to have high pro-inflammatory responses to control infection, which also results in the clinical manifestation of disease. With age, due to multiple exposure to infection, the immune system is perhaps trained and the balance shifts to a predominance of anti-inflammatory responses to control the infection with no clinical signs of disease, to a point that infection is tolerated and parasitemia is maintained. Since immunity to malaria is not sterile, there appears to be a trade-off between anti-parasite and anti-disease immunity, with the latter dominating in asymptomatic infections, where the focus is controlling disease rather than clearing parasites. This compromise in host immunity to control disease and not clear parasites is likely driven by the anti-inflammatory immune response. A transcriptomic approach to analyzing the cells and cytokines involved in the process would provide the necessary insight to unraveling the role of both anti- and pro-inflammatory responses to asymptomatic infections.

Though there is still a gap in our understanding of how the parasite remains in an asymptomatic state in individuals from malaria endemic areas, various studies have implicated genes responsible for gene regulation and chromatin remodeling and it is still unclear how host immunity is involved. High-throughput studies are needed to understand differential gene expression in immune cells that have shown differential activity in asymptomatic vs. symptomatic or uninfected states (5). Recently, single cell transcriptomics has emerged as a new approach in transcriptomic studies that characterizes individual cells, improving our ability to study cell to cell variability, unlike the conventional transcriptome method that assumes and treats cells from a certain tissue as homogenous (110). Reid et al. (111) used this technique to show transcription variation across all stages of the parasite life cycle and how genes involved in immune evasion aid the parasite in transiting from the asexual stage in humans to its sexual stage in the mosquito (111). Properly designed asymptomatic *P. falciparum* single cell RNA-seq studies of individual immune cells and parasites with good criteria for defining asymptomatic infections hold the key to understanding these chronic and debilitating infections as well as host pathogen interactions in general.

Furthermore, there is a need for a consensus on how asymptomatic infections are defined. Longitudinal cohorts or follow ups over a period of 6 weeks, 4 weeks prior to the asymptomatic case and 2 weeks following the case, with no history or evidence of the individual having taken antimalarials and no evidence of fever 48 h before and after the case, are likely to be the most feasible approach to defining an asymptomatic infection. More sensitive tools for defining the presence of parasitemia to minimize missing sub-patent infections requires methods such as PCR, LAMP, or the newly described hemozoin sensing assay (33). With a clear definition of asymptomatic infections, further downstream analyses become possible. Exploiting next generation sequencing platforms to conduct amplicon deep sequencing and RNA-Seq has the potential to allow for a comprehensive analysis of asymptomatic infections to a scale not previously explored. The scale to which COI can be defined using amplicon deep sequencing improves the accuracy of determining COI in asymptomatic infections, the impact of COI on the risk of developing febrile disease and immunity. RNA-seq promises to unravel the complexities of the host immune response to malaria infection, describing at the transcriptomic level which molecules are likely to be involved in particular processes of controlling infection or, in the case of asymptomatic infections, maintaining parasitemia with no clinical symptoms. Of interest in this review was the role of anti- and pro-inflammatory cytokines in determining the course of infection. A focused analysis of transcripts related to these pathways would provide a better understanding of the role cytokines play in regulating the immune system and influencing malaria outcome. Notably, a better understanding of chromatin remodeling pathways is required to determine whether they are associated with particular gene transcripts, and ATAC-Seq will allow further interrogation of these pathways.

## Author Contributions

KK and LO-O conceived, drafted, and reviewed the manuscript. KW drafted and reviewed the manuscript.

### Conflict of Interest

The authors declare that the research was conducted in the absence of any commercial or financial relationships that could be construed as a potential conflict of interest.
